# Hybrid BFPSO algorithm based estimation of optimal drug dosage for antiretroviral therapy in HIV-1 infected patients

**DOI:** 10.1186/1471-2334-14-S3-E14

**Published:** 2014-05-27

**Authors:** K Kamalanand, P Mannar Jawahar

**Affiliations:** 1 Madras Institute of Technology Campus, Anna University, Chennai, India

## Background

Although the use of anti retroviral drugs has prolonged the life of infected patients, the side effects can be severe. The objective of this work is to efficiently estimate the optimal drug dosage for ART using mathematical model based method.

## Methods

In this work, an artificial intelligence technique known as the hybrid Bacterial Foraging Particle Swarm Optimization (BFPSO) algorithm is used in conjunction with the three dimensional patient specific HIV model, for estimation of optimal drug concentration. In the mathematical model, it is assumed that the drug acts directly on the viral burden. The optimization problem is formulated in such a way that the HIV-1 viral load as well as the total drug concentration is minimized.

## Results

Based on the computer simulation studies performed for a particular case at the seventh year of infection, it was found that in the scenario without therapy, the CD4 cell count dropped to a value of 366 cells/mm^3^. However, when ART is administered based on the hybrid BFPSO algorithm, the CD4 cell count increased to a value of 782 cells/mm^3^, with minimal usage of the drug concentration.

## Conclusion

Results demonstrate that, the optimal drug dosage estimated using the proposed methodology is efficient in improving the immune system and minimizing the viral burden. This work seems to be of high clinical importance since, at present; ART is the widely used method for treatment of HIV-1 infection.

